# Decoding the Bone–Eye Axis: Machine Learning for Age-Related Macular Degeneration Risk Prediction

**DOI:** 10.34133/cbsystems.0676

**Published:** 2026-09-14

**Authors:** Xuehao Cui, Qiuchen Zhao, Jingwen Hui, Zheya Han, Patrick Yu-Wai-Man, Quanhong Han

**Affiliations:** ^1^ Department of Clinical Neurosciences, University of Cambridge, Cambridge CB2 0XY, UK.; ^2^ John van Geest Centre for Brain Repair, University of Cambridge, Cambridge CB2 0PY, UK.; ^3^ Cambridge Eye Unit, Addenbrooke’s Hospital, Cambridge University Hospitals NHS Foundation Trust, Cambridge CB2 0QQ, UK.; ^4^ Department of Ophthalmology, Yale University, New Haven, CT 06520, USA.; ^5^ Tianjin Eye Hospital, Tianjin Key Lab of Ophthalmology and Visual Science, Nankai University, Tianjin 300020, China.

## Abstract

Age-related macular degeneration (AMD) is a leading cause of irreversible vision loss, yet systemic determinants of its risk remain incompletely understood. Bone mineral density (BMD), a marker of skeletal and biological aging, may reflect shared pathways linking systemic and retinal degeneration. We investigated the association between BMD and AMD using a multilayered framework integrating epidemiological analyses, Mendelian randomization (MR), proteomic and metabolomic profiling, machine learning, and an experimental low-BMD model. Across 3 cohorts, including UK Biobank, National Health and Nutrition Examination Survey, and a hospital-based Tianjin cohort, lower BMD was consistently associated with higher AMD risk, although AMD ascertainment and BMD measurement differed across cohorts. Two-sample MR analyses provided supportive genetic evidence consistent with a modest potential contribution of higher BMD to lower AMD risk, while sensitivity analyses did not indicate strong evidence of reverse causality. Machine learning models identified BMD as a recurrent predictive contributor alongside age, but its incremental clinical utility requires formal evaluation using models with and without BMD. UK Biobank proteomic and metabolomic analyses revealed overlapping molecular signatures involving extracellular matrix remodeling, lipid transport, amino acid metabolism, and inflammatory pathways. Two-step MR prioritized circulating proteins including granzyme A, collagen type II alpha 1 chain, and NEL-like protein 1 as candidate molecular intermediates rather than established mechanistic mediators. In a glucocorticoid-induced low-BMD rat model, retinal alterations relevant to degeneration, including outer retinal thinning, vascular narrowing, and delayed visual–spatial performance, were observed, but these findings should not be interpreted as direct validation of AMD pathology. Overall, these findings support an association between lower BMD and AMD risk and suggest that BMD may act as an accessible marker of systemic aging processes related to retinal vulnerability.

## Introduction

Age-related macular degeneration (AMD) is a leading cause of irreversible central vision loss in older adults, accounting for approximately 9% of global blindness [1–3]. With population aging, its burden continues to rise, affecting ~200 million people worldwide in 2020 and projected to reach nearly 300 million by 2040 [4,5]. Early AMD is characterized by drusen deposition and retinal pigment epithelium dysfunction, whereas advanced disease manifests as neovascular or atrophic forms [6,7]. Neovascular AMD involves choroidal neovascularization with exudation and hemorrhage, while atrophic AMD is marked by progressive retinal degeneration and geographic atrophy [8–10]. Despite differing clinical courses, both ultimately lead to central vision loss and substantial impacts on independence and quality of life [11]. The heterogeneity and multistage progression of AMD highlight the importance of early identification and risk stratification [12].

Bone mineral density (BMD) is a key indicator of skeletal strength and a marker of systemic aging [13–15]. Its decline underlies osteoporosis and increased fracture risk [16], affecting up to one in 3 women and one in 5 men over 50 years of age [17]. Beyond skeletal structure, BMD reflects the integrated influence of endocrine, metabolic, and immune processes [18,19], including hormonal regulation, mineral homeostasis, inflammation, and lifestyle factors [20,21]. The skeleton also functions as an endocrine organ, influencing distant tissues and supporting the concept of BMD as a systemic biomarker [22].

Emerging evidence suggests a potential “bone–eye axis” linking skeletal health and AMD. Shared mechanisms include chronic low-grade inflammation [23], oxidative stress, mitochondrial dysfunction, and microvascular alterations, all of which may simultaneously affect bone and retinal integrity [24–26]. Disturbances in lipid metabolism, extracellular matrix turnover, and complement activation further support this overlap [27,28]. However, existing epidemiological evidence remains inconsistent and limited, largely based on cross-sectional, single-population studies with small sample sizes, precluding robust inference on causality and generalizability [29]. Moreover, molecular mechanisms underlying this association remain poorly characterized, and integrative multiomics approaches have rarely been applied.

To address these gaps, we established a multinational, multidimensional framework integrating large-scale epidemiological data, multiomics profiling, and machine learning. This approach aims to (a) provide robust population-level evidence; (b) identify potential biological pathways; and (c) evaluate the incremental predictive value of BMD for AMD risk stratification. In addition, a low-BMD rat model was used to examine retinal structural and functional alterations, bridging population findings with experimental evidence. By integrating epidemiological breadth, multiomics depth, and computational modeling, this study seeks to refine the understanding of the bone–eye axis and provide a basis for future studies of AMD risk stratification and systemic-aging-related mechanisms.

## Materials and Methods

### Study design and populations

#### Analytical framework

**Fig. 1.**
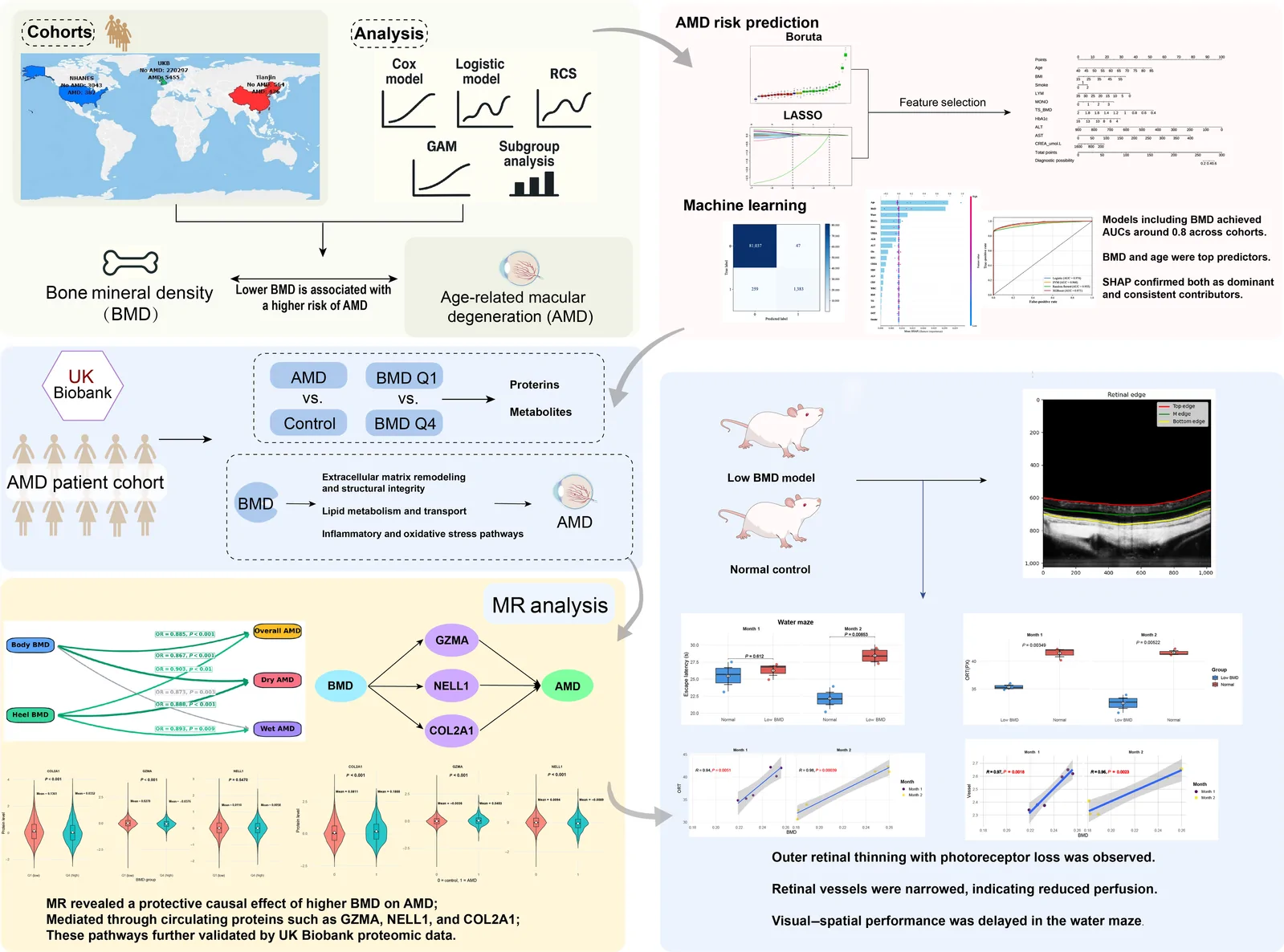
Study design. Overview of the multinational and multidimensional framework integrating 3 human cohorts (UK Biobank, National Health and Nutrition Examination Survey [NHANES], and Tianjin), 2-sample Mendelian randomization, proteomic and metabolomic analyses, machine learning models, and a low-bone-density rat model to investigate the bone–eye axis between bone mineral density (BMD) and age-related macular degeneration (AMD). OR, odds ratio; SVM, support vector machine; ORT, outer retina thickness.

This study combined multicohort observational analyses with 2-sample Mendelian randomization (MR), multiomics profiling, machine learning modeling, and experimental validation (Fig. 1). Three independent datasets with different designs were included. UK Biobank (UKB) provided a large prospective population-based cohort with longitudinal follow-up, National Health and Nutrition Examination Survey (NHANES) 2005–2008 provided a cross-sectional population survey with retinal imaging, and the Tianjin dataset provided a hospital-based clinical case-control cohort. Because the Tianjin cohort had a much higher AMD case proportion than expected in the general population, it was interpreted as hospital-based replication rather than population-level validation.

#### Cohort-specific AMD ascertainment

AMD ascertainment was cohort specific. UKB captured AMD using medical records and self-report and therefore had limited resolution for early, intermediate, geographic atrophy, neovascular, wet, or dry AMD categories. NHANES provided imaging-based ascertainment, whereas the Tianjin cohort used ophthalmologist-confirmed clinical diagnosis. AMD phenotype definitions were not assumed to represent identical disease-stage distributions.

#### Cohort-specific BMD assessment

BMD assessment was also cohort specific. In UKB, BMD was assessed using heel-ultrasound-derived estimated BMD and analyzed as a standardized BMD score. In NHANES, BMD was measured by dual-energy x-ray absorptiometry (DXA), with cohort-specific units retained and standardized before regression modeling. In the Tianjin cohort, BMD was measured using clinical DXA.

#### Cross-cohort interpretation

The datasets differed in diagnostic modality, disease-stage resolution, skeletal site, measurement principle, and error structure. They were therefore treated as complementary sources of evidence rather than direct replications of a single harmonized end point. Cross-cohort comparisons emphasized the direction and consistency of associations rather than direct equivalence. Outcome misclassification was expected to be greatest in UKB because record-based and self-reported AMD may miss early or asymptomatic disease. This would most likely attenuate population-level associations, although differential healthcare contact may also introduce bias.

### Observational analyses

#### Baseline comparisons and association models

Baseline characteristics were compared using Student’s *t* test or Wilcoxon rank sum test for continuous variables and chi-square tests for categorical variables. Associations between BMD and AMD were evaluated using Cox proportional hazards models in UKB and multivariable logistic regression models in NHANES and Tianjin. BMD was analyzed both as a continuous variable and categorized into quartiles (Q1 to Q4).

#### Covariate adjustment

Three models were constructed with increasing levels of adjustment. Model 1 adjusted for age, sex, and ethnicity where available. Model 2 additionally adjusted for body mass index (BMI), waist circumference, smoking status, alcohol consumption, hypertension, diabetes, and education level where available. Model 3 further included cohort-specific biochemical variables. In UKB, model 3 included fasting glucose, hemoglobin A1c (HbA1c), red blood cell count, white blood cell count, platelet count, C-reactive protein, albumin, alkaline phosphatase, alanine aminotransferase, aspartate aminotransferase, gamma-glutamyl transferase, creatinine, urea, urate, total cholesterol, and triglycerides. In NHANES, model 3 included HbA1c, red blood cell count, albumin, alkaline phosphatase, alanine aminotransferase, aspartate aminotransferase, gamma-glutamyl transferase, creatinine, urate, total cholesterol, high-density lipoprotein (HDL) cholesterol, and poverty income ratio. In the Tianjin cohort, model 3 included HbA1c, red blood cell count, albumin, alkaline phosphatase, alanine aminotransferase, aspartate aminotransferase, gamma-glutamyl transferase, creatinine, urate, total cholesterol, HDL cholesterol, lymphocyte count, monocyte count, and neutrophil count. Because several biochemical variables may represent confounders, downstream consequences, or intermediate systemic markers linking BMD and AMD, model 3 was interpreted as a broadly adjusted association model rather than as a purely confounding-controlled causal estimate. Missing data were handled within each cohort before modeling. Continuous variables with missing values were imputed using cohort-specific medians, and categorical variables were imputed using cohort-specific modes unless otherwise stated. Variables included in model 3 were selected on the basis of availability, biological plausibility, and consistency with baseline clinical indicators rather than data-driven outcome selection. We recognized that some biochemical variables may lie downstream of BMD-related systemic aging processes or represent intermediate markers linking skeletal and retinal degeneration. Therefore, model 3 was used as a sensitivity analysis for broad systemic adjustment, whereas model 2 was treated as the main multivariable model for confounder adjustment. The causal roles of inflammatory, metabolic, renal, hepatic, and lipid-related variables were interpreted cautiously within a predefined conceptual framework rather than assumed to be pure confounders.

#### Nonlinear and subgroup analyses

Nonlinear associations were assessed using restricted cubic spline (RCS) functions with knots placed at the 5th, 35th, 65th, and 95th percentiles, as well as generalized additive models (GAMs) with smoothing splines. Subgroup analyses were conducted stratified by sex, smoking status, alcohol consumption, hypertension, and diabetes, with interaction tests performed where appropriate.

### Feature selection and risk prediction model

Feature selection was performed using Boruta and least absolute shrinkage and selection operator (LASSO) regression [30]. The Boruta algorithm was implemented on the basis of random forest models with 500 iterations to identify relevant variables [31]. LASSO regression was performed with 10-fold cross-validation, and the optimal penalty parameter (*λ*) was selected on the basis of minimum deviance [32]. Variables consistently selected by both methods were used to construct multivariable logistic regression models and nomograms. Model performance was evaluated using receiver operating characteristic (ROC) curves, area under the curve (AUC), and calibration analysis.

### Machine learning classification

#### Algorithms and hyperparameter tuning

Four machine learning algorithms were applied: regularized logistic regression, support vector machine with a radial basis function kernel, random forest, and extreme gradient boosting (XGBoost). Hyperparameters were tuned within the training set using cross-validation. For logistic regression, the regularization strength was selected from candidate values of *C*. For support vector machine, the cost parameter and kernel coefficient gamma were tuned. For random forest, the number of trees, number of variables sampled at each split, and minimum terminal node size were evaluated. For XGBoost, maximum tree depth, learning rate, number of boosting rounds, subsampling fraction, and column sampling fraction were tuned [32].

#### Data splitting and internal validation

For each cohort, models were developed using cohort-specific training and testing procedures. In NHANES and Tianjin, participants were divided into training (70%) and testing (30%) sets using stratified sampling to preserve the AMD case proportion. Within the training set, 5-fold cross-validation was used for hyperparameter tuning and model selection, with a fixed random seed of 2026. Reported test set AUCs were calculated only in the held-out testing set. In UKB, stratified sampling was used, and performance was evaluated using held-out testing data after preprocessing and tuning within the training set.

#### Class imbalance and performance assessment

Because AMD cases were uncommon in UKB, class weighting was applied and evaluation was not based on accuracy alone. Performance was reported using AUC, sensitivity, specificity, positive predictive value, negative predictive value, F1 score, and confusion matrices. Precision–recall curves and threshold sensitivity analyses were additionally used in UKB to assess model behavior under low-prevalence conditions.

#### Leakage prevention and model interpretation

To prevent data leakage, missing value imputation, variable scaling, feature selection, and hyperparameter tuning were performed exclusively within the training set. Learned parameters were applied unchanged to the held-out test set, which was not used for feature selection, model fitting, threshold optimization, or tuning. Model interpretability was assessed using SHapley Additive exPlanation (SHAP), with mean absolute SHAP values used to rank predictive contributions and visualize nonlinear effects. The full cohort-specific workflow is summarized in Table S22.

### MR analysis

Two-sample MR analyses were performed using publicly available genome-wide association study (GWAS) summary statistics for total body BMD, heel BMD, and AMD. Single-nucleotide polymorphisms (SNPs) associated with BMD at genome-wide significance were clumped for linkage disequilibrium using *r*^2^ < 0.001 within a 10,000-kb window. Weak instrument bias was assessed using *F* statistics, and SNPs with *F* statistics ≤10 were excluded. Directionality was evaluated using Steiger filtering. Horizontal pleiotropy and outlier instruments were assessed using MR-Egger intercept testing, Cochran’s *Q* statistic, leave-one-out analysis, and MR-PRESSO (Pleiotropy RESidual Sum and Outlier) where applicable. Because BMD and AMD are genetically complex traits and may share pleiotropic pathways with BMI, lipids, vitamin D, and inflammation, MR estimates were interpreted as supportive genetic evidence rather than definitive causal proof. Detailed GWAS data sources, including total body BMD, heel BMD, overall AMD, dry AMD, wet AMD, circulating protein quantitative trait loci (pQTLs), metabolite GWAS, and cardiometabolic GWAS datasets used in multivariable MR, are summarized in Table S21.

Primary causal estimates were obtained using the inverse-variance weighted (IVW) method under a random-effects model. Sensitivity analyses were conducted using complementary approaches, including weighted median, weighted mode, and MR-Egger regression, to assess robustness under different assumptions regarding invalid instruments. Horizontal pleiotropy was evaluated using the MR-Egger intercept test, and heterogeneity across instruments was assessed using Cochran’s *Q* statistic. Leave-one-out analyses were further performed to determine whether results were driven by individual SNPs.

To account for potential confounding by metabolic traits, multivariable MR analyses were conducted by jointly modeling BMD with BMI, fasting glucose, total cholesterol, triglycerides, and circulating vitamin D levels. In addition, a 2-step mediation MR framework was implemented to identify potential protein mediators linking BMD to AMD. In the first step, BMD-associated SNPs were used to estimate causal effects on circulating protein levels using pQTL data. In the second step, protein-associated SNP effects were evaluated against AMD risk. Only proteins with consistent directionality across both steps were considered candidate mediators.

### Proteomic and metabolomic analyses

Proteomic and metabolomic analyses were conducted using the UKB subset with available multiomics data. Plasma proteomic profiling was performed using the Olink platform (proximity extension assay), covering a wide range of inflammation, cardiovascular, and metabolic proteins. Metabolomic profiling was based on nuclear magnetic resonance spectroscopy, quantifying lipid fractions, amino acids, and other small-molecule metabolites.

Quality control procedures included exclusion of samples with missing values exceeding predefined thresholds, normalization of protein expression levels, and log transformation where appropriate. Participants were stratified into BMD quartiles, and differential analyses were conducted comparing individuals in the lowest (Q1) and highest (Q4) BMD groups using linear or logistic regression models adjusted for age, sex, and relevant covariates.

Associations between identified proteins/metabolites and AMD were evaluated using logistic regression for cross-sectional outcomes and Cox proportional hazards models for incident AMD in UKB. Molecules importantly associated with both BMD and AMD after false discovery rate (FDR) correction were considered overlapping molecular signatures. Cross-sectional proteomic and metabolomic associations were interpreted as shared biomarkers or pathway-level signals rather than direct evidence of mechanistic mediation. Only molecules supported by directionally consistent 2-step MR analyses were described as candidate molecular intermediates, and these were not considered established mediators unless supported by additional temporal or experimental evidence.

Functional pathway enrichment analysis was performed using the Kyoto Encyclopedia of Genes and Genomes (KEGG) database. Enrichment significance was assessed using hypergeometric testing, and multiple comparisons were corrected using the Benjamini–Hochberg FDR method.

### Pathway enrichment analysis

To gain further insight into the implicated biological pathways, we performed functional pathway enrichment analysis on the metabolites identified in the metabolomic analysis. Specifically, we conducted an enrichment analysis using the KEGG database [33]. Metabolites were grouped according to predefined statistical criteria: BMD-associated metabolites were defined as those associated with BMD after FDR correction, and AMD-associated metabolites were defined as those associated with AMD at the prespecified significance threshold. KEGG enrichment was then performed separately for each metabolite set using the successfully mapped metabolites as the input list and all annotated metabolites in the analytic metabolomic dataset as the background set. This allowed us to identify metabolic pathways that were overrepresented in these sets of metabolites. By examining the overlapping or intersecting pathways, we could infer potential metabolic pathways through which BMD may influence AMD risk.

Metabolite identifiers were mapped to KEGG compound IDs before enrichment analysis. The mapping rate, unmapped metabolites, duplicated mappings, and final number of annotated metabolites were recorded. When multiple metabolomic features mapped to the same KEGG compound, duplicated entries were collapsed to avoid overweighting the same compound. Unannotated metabolites and lipoprotein subclass features without valid KEGG compound IDs were excluded from KEGG enrichment but retained in descriptive metabolomic analyses. The enrichment background was defined as all KEGG-annotated metabolites available in the analytic metabolomic dataset. Enrichment was tested using hypergeometric tests, and *P* values were corrected using the Benjamini–Hochberg FDR method. The enrichment ratio was calculated as the proportion of input metabolites annotated to a given pathway divided by the corresponding proportion in the background set.

### Animal experiment

All animal experiments were conducted in accordance with institutional guidelines and approved by the local Animal Care and Use Committee. Female Sprague–Dawley rats (12 weeks old, 200 to 250 g) were obtained from an accredited animal facility and housed under controlled conditions (22 °C ± 2 °C, 12-h light/dark cycle, free access to food and water).

Rats were randomly assigned to a low-BMD group or control group (*n* = 3 per group). Low BMD was induced by intraperitoneal injection of prednisolone at a dose of 6 mg/kg per day for 4 consecutive weeks, a widely established model of glucocorticoid-induced osteoporosis. Control animals received equivalent volumes of sterile saline.

At 4 and 8 weeks following model induction, animals underwent retinal imaging and functional testing. For imaging, rats were anesthetized using intraperitoneal pentobarbital sodium (40 mg/kg), and pupils were dilated with topical tropicamide (0.5%). Retinal structure was assessed using spectral domain optical coherence tomography (Heidelberg Engineering), and fundus images were acquired using a Micron IV retinal imaging system (Phoenix Research Labs). Retinal thickness parameters, including total retinal thickness, inner retinal thickness, and outer retinal thickness, were measured on the basis of standardized layer segmentation. Retinal vessel caliber was quantified from fundus images using ImageJ software with the VesselJ plugin, following binarization, skeletonization, and vessel diameter extraction procedures.

Visual–spatial and behavioral performance was assessed using the Morris water maze test. Rats were trained to locate a hidden platform in a circular pool using visual cues over multiple trials. Escape latency was recorded as a behavioral performance measure influenced by visual–spatial processing, learning, motor activity, stress response, and systemic health, rather than as a direct or isolated measure of visual impairment. All image acquisition and quantitative analyses were performed by investigators blinded to group allocation.

BMD was measured at each time point to confirm successful model induction. Correlation analyses were conducted between BMD values and retinal structural parameters.

### Statistical analysis and software

All statistical analyses were performed using R (version 4.2.2) and Python (version 3.9). Regression analyses were conducted using standard packages including survival, glm, and mgcv. Machine learning analyses were implemented using scikit-learn and XGBoost. MR analyses were performed using the TwoSampleMR package. Continuous variables are presented as means ± SD, and categorical variables are presented as counts and percentages. Two-sided *P* values of <0.05 were considered statistically significant. Multiple testing correction was applied using the Benjamini–Hochberg FDR where appropriate.

### Mathematical formulations of statistical and computational methods

To improve methodological transparency, the main statistical, machine learning, MR, and enrichment analyses were described using the following model expressions. For all models, i indexes participants, ${\mathrm{BMD}}_{i}$ denotes BMD, $X_{i}$ denotes a vector of covariates, and β denotes the corresponding regression coefficients. Continuous predictors were standardized within each cohort before modeling when required by the algorithm:

$$Z_{\mathrm{ij}}=\frac{X_{\mathrm{ij}}-\mu_{j}}{\sigma_{j}}$$ (1)

where $X_{\mathrm{ij}}$ is the original value of variable j for participant i, $\mu_{j}$ is the cohort-specific mean, and $\sigma_{j}$ is the cohort-specific SD.

For prospective analyses in UKB, Cox proportional hazards models were used to estimate the association between baseline BMD and incident AMD:

$$h_{i}\left(t|X_{i}\right)=h_{0}\left(t\right)\exp\left(\beta_{1}{\mathrm{BMD}}_{i}+\beta^{⊤}X_{i}\right)$$ (2)

where $h_{i}\left(t|X_{i}\right)$ is the hazard of incident AMD at time t; $h_{0}\left(t\right)$ is the baseline hazard; $\beta_{1}$ is the coefficient for BMD; and $X_{i}$ includes demographic, lifestyle, anthropometric, and biochemical covariates according to the model specification. For cross-sectional analyses in NHANES and the Tianjin cohort, multivariable logistic regression models were specified as:

$$\text{logit}\left(p_{i}\right)=\log\left(\frac{p_{i}}{1-p_{i}}\right)=\beta_{0}+\beta_{1}{\mathrm{BMD}}_{i}+\beta^{⊤}X_{i}$$ (3)

where $p_{i}=\mathrm{Pr}\left(Y_{i}=1|{\mathrm{BMD}}_{i},X_{i}\right)$ is the probability of AMD.

For quartile-based analyses, BMD quartiles were entered as categorical indicator variables using the lowest quartile as the reference group:

$$\text{logit}\left(p_{i}\right)=\beta_{0}+\beta_{2}Q_{2i}+\beta_{3}Q_{3i}+\beta_{4}Q_{4i}+\beta^{⊤}X_{i}$$ (4)

where $Q_{2i}$, $Q_{3i}$, and $Q_{4i}$ indicate membership in the second, third, and fourth BMD quartiles, respectively.

Nonlinear associations were evaluated using RCS functions with knots placed at the 5th, 35th, 65th, and 95th percentiles of BMD:

$$g\left\{E\left(Y_{i}\right)\right\}=\beta_{0}+f_{\mathrm{RCS}}\left({\mathrm{BMD}}_{i}\right)+\beta^{⊤}X_{i}$$ (5)

where $g\left(\cdot \right)$ is the appropriate link function and $f_{\mathrm{RCS}}\left(\cdot \right)$ denotes the RCS transformation of BMD. GAMs were additionally fitted as:

$$g\left\{E\left(Y_{i}\right)\right\}=\beta_{0}+s\left({\mathrm{BMD}}_{i}\right)+\beta^{⊤}X_{i}$$ (6)

where $s\left(\cdot \right)$ is a smooth function estimated from the data. Subgroup interactions were evaluated by including multiplicative interaction terms:

$$g\left\{E\left(Y_{i}\right)\right\}=\beta_{0}+\beta_{1}{\mathrm{BMD}}_{i}+\beta_{2}G_{i}+\beta_{3}\left({\mathrm{BMD}}_{i}\cdot G_{i}\right)+\beta^{⊤}X_{i}$$ (7)

where $G_{i}$ denotes the subgroup indicator and the coefficient $\beta_{3}$ tests for effect modification.

For feature selection, LASSO regression minimized the penalized objective function:

$$\hat{\beta }=\arg\min_{\beta }\left\{-\ell \left(\beta \right)+\lambda \sum_{j=1}^{p}\left|\beta_{j}\right|\right\}$$ (8)

where $\ell \left(\beta \right)$ denotes the log likelihood, λ controls the degree of shrinkage, and p is the number of candidate predictors. The optimal λ was selected using cross-validation. Boruta feature selection was implemented using random forest-derived importance scores by comparing observed variable importance with the importance of randomly permuted shadow variables.

For machine learning classification, regularized logistic regression estimated individual AMD probability as:

$${\hat{p}}_{i}=\frac{1}{1+\exp\left[-\left(\beta_{0}+\sum_{j=1}^{p}\beta_{j}x_{\mathrm{ij}}\right)\right]}$$ (9)

Support vector machine models with a radial basis function kernel used the kernel function:

$$K\left(x_{i},x_{i\prime }\right)=\exp\left(-\gamma ∥x_{i}-x_{i\prime }{∥}^{2}\right)$$ (10)

and the classification function:

$$f\left(x\right)=\mathrm{sign}\left(\sum_{i=1}^{n}\alpha_{i}y_{i}K\left(x_{i},x\right)+b\right)$$ (11)

where γ is the kernel coefficient, $\alpha_{i}$ are support vector weights, $y_{i}$ is the class label, and b is the intercept. Random forest predictions were obtained by averaging predictions from T decision trees:

$${\hat{p}}_{i}=\frac{1}{T}\sum_{t=1}^{T}{\hat{p}}_{\mathrm{it}}^{\left(t\right)}$$ (12)

XGBoost was formulated as an additive tree ensemble:

$${\hat{y}}_{i}=\sum_{m=1}^{M}f_{m}\left(x_{i}\right),\;f_{m}\in F$$ (13)

with the regularized objective function:

$$L=\sum_{i=1}^{n}l\left(y_{i},{\hat{y}}_{i}\right)+\sum_{m=1}^{M}\Omega \left(f_{m}\right)$$ (14)

where $l\left(y_{i},{\hat{y}}_{i}\right)$ is the prediction loss and $\Omega \left(f_{m}\right)$ penalizes model complexity.

Model discrimination and classification performance were assessed using AUC, accuracy (Acc), sensitivity (Sens), specificity (Spec), positive predictive value (PPV), negative predictive value (NPV), F1 score, and calibration metrics. These metrics were defined as:

$$\mathrm{Acc}=\frac{\mathrm{TP}+\mathrm{TN}}{\mathrm{TP}+\mathrm{TN}+\mathrm{FP}+\mathrm{FN}}$$ (15)

$$\text{Sens}=\frac{\mathrm{TP}}{\mathrm{TP}+\mathrm{FN}},\;\text{Spec}=\frac{\mathrm{TN}}{\mathrm{TN}+\mathrm{FP}}$$ (16)

$$\mathrm{PPV}=\frac{\mathrm{TP}}{\mathrm{TP}+\mathrm{FP}},\;\mathrm{NPV}=\frac{\mathrm{TN}}{\mathrm{TN}+\mathrm{FN}}$$ (17)

$$F_{1}=\frac{2\mathrm{PPV}\cdot \text{Sens}}{\mathrm{PPV}\cdot \text{Sens}}$$ (18)

$$\mathrm{BS}=\frac{1}{n}\sum_{i=1}^{n}{\left(y_{i}-{\hat{p}}_{i}\right)}^{2}$$ (19)

SHAP values were used to quantify model-based predictive contributions. For a trained model f, the prediction for participant i was decomposed as:

$$f\left(x_{i}\right)=\phi_{0}+\sum_{j=1}^{p}\phi_{\mathrm{ij}}$$ (20)

where $\phi_{0}$ is the baseline prediction and $\phi_{\mathrm{ij}}$ is the SHAP contribution of feature j for participant i. The Shapley value for feature j is defined as:

$$\begin{array}{c} \phi_{j}=\sum_{S\subseteq F_{j}}\frac{\left|S\right|!\left(p-\left|S\right|-1\right)!}{p!}\left[\;f\left(S,j\right)-f\left(S\right)\right] \end{array}$$ (21)

where $F_{j}$ denotes the full feature set excluding feature j, S is a subset of $F_{j}$, and $f\left(S,j\right)-f\left(S\right)$ represents the change in model prediction after adding feature j to subset S. SHAP values were interpreted as predictive contributions rather than causal effects.

For MR, the SNP-specific Wald ratio estimate was calculated as:

$${\hat{\theta }}_{j}=\frac{{\hat{\beta }}_{\mathrm{Yj}}}{{\hat{\beta }}_{\mathrm{Xj}}}$$ (22)

where ${\hat{\beta }}_{\mathrm{Xj}}$ is the SNP-exposure association and ${\hat{\beta }}_{\mathrm{Yj}}$ is the SNP outcome association for genetic variant j. Instrument strength was assessed using the *F* statistics:

$$F_{j}=\frac{{\hat{\beta }}_{\mathrm{Xj}}^{2}}{\mathrm{SE}{\left({\hat{\beta }}_{\mathrm{Xj}}\right)}^{2}}$$ (23)

The primary IVW estimate was obtained as:

$${\hat{\theta }}_{\mathrm{IVW}}=\frac{\sum_{j=1}^{K}w_{j}{\hat{\theta }}_{j}}{\sum_{j=1}^{K}w_{j}}$$ (24)

where K is the number of genetic instruments and $w_{j}=1/\mathrm{SE}{\left({\hat{\theta }}_{j}\right)}^{2}$ is the inverse-variance weight. MR-Egger regression was specified as:

$${\hat{\beta }}_{\mathrm{Yj}}=\alpha +\theta_{\text{Egger}}{\hat{\beta }}_{\mathrm{Xj}}+\varepsilon_{j}$$ (25)

where the intercept α was used to assess directional horizontal pleiotropy. Heterogeneity across instruments was assessed using Cochran’s *Q* statistic:

$$Q=\sum_{j=1}^{K}w_{j}{\left({\hat{\theta }}_{j}-{\hat{\theta }}_{\mathrm{IVW}}\right)}^{2}$$ (26)

For multivariable MR, SNP outcome associations were regressed on SNP associations with BMD and other cardiometabolic traits:

$${\hat{\beta }}_{\mathrm{Yj}}=\theta_{1}{\hat{\beta }}_{\mathrm{BMD},j}+\theta_{2}{\hat{\beta }}_{\mathrm{BMI},j}+\theta_{3}{\hat{\beta }}_{\text{glucose},j}+\theta_{4}{\hat{\beta }}_{\text{lipid},j}+\theta_{5}{\hat{\beta }}_{\text{vitaminD},j}+\varepsilon_{j}$$ (27)

For 2-step mediation MR, the indirect effect through a candidate mediator M was estimated as:

$$\theta_{\text{indirect}}=\theta_{\mathrm{BMD}\to M}\cdot \theta_{M\to \mathrm{AMD}}$$ (28)

and the proportion mediated (PM) was calculated as:

$$\mathrm{PM}=\frac{\theta_{\text{indirect}}}{\theta_{\text{total}}}$$ (29)

Proteomic and metabolomic association analyses were modeled using regression frameworks appropriate for continuous molecular traits and binary AMD status. For each molecular feature $M_{i}$, the BMD association model was specified as:

$$M_{i}=\alpha_{0}+\alpha_{1}{\mathrm{BMD}}_{i}+\alpha^{⊤}X_{i}+\varepsilon_{i}$$ (30)

For molecular associations with AMD status, logistic regression was used:

$$\text{logit}\left\{\mathrm{Pr}\left({\mathrm{AMD}}_{i}=1\right)\right\}=\delta_{0}+\delta_{1}M_{i}+\delta^{⊤}X_{i}$$ (31)

For incident AMD in UKB, molecular features were additionally evaluated using Cox models:

$$h_{i}\left(t|M_{i},X_{i}\right)=h_{0}\left(t\right)\exp\left(\delta_{1}M_{i}+\delta^{⊤}X_{i}\right)$$ (32)

Multiple testing was controlled using the Benjamini–Hochberg FDR procedure. For ordered P values $p_{\left(1\right)}\leq p_{\left(2\right)}\leq \cdots \leq p_{\left(m\right)}$, the adjusted value was calculated as:

$$q_{\left(i\right)}=\min_{j\geq i}\left\{\frac{m}{j}p_{\left(j\right)}\right\}$$ (33)

For KEGG pathway enrichment analysis, enrichment significance was assessed using a hypergeometric test:

P=∑x=kminK,nKxN−Kn−xNn (34)

where N is the number of annotated metabolites in the background set, K is the number of background metabolites annotated to a given pathway, n is the number of input metabolites, and k is the number of input metabolites annotated to that pathway. The enrichment ratio was defined as:

$$\mathrm{Enrichment}\;\mathrm{Ratio}=\frac{k/n}{K/N}$$ (35)

Together, these mathematical expressions were added to clarify the modeling assumptions, objective functions, performance metrics, and interpretation of statistical, machine learning, MR, omics, and enrichment analyses.

## Results

### Baseline characteristics

The analytic cohorts included 270,297 participants without AMD and 5,455 with AMD in UKB, 3,043 without AMD and 382 with AMD in NHANES, and 564 without AMD and 436 with AMD in Tianjin (Table 1). Across all 3 cohorts, participants with AMD were older and had lower BMD than controls, underscoring age as a major confounder and biological context for the observed BMD–AMD association. In UKB, the mean age was 62.72 years in cases versus 56.31 years in controls; corresponding values were 68.70 versus 56.30 years in NHANES and 67.36 versus 58.31 years in Tianjin (all *P* < 0.001). BMD showed the same pattern. In UKB, the mean BMD *z*-score was −0.19 in cases and 0.02 in controls; in NHANES, mean hip BMD was 0.94 versus 1.03; and in Tianjin, mean BMD was 0.86 versus 1.00 (all *P* < 0.001).

**Table 1.** Summary table of baseline characteristics and clinical indicators in the UKB, NHANES, and Tianjin cohorts

Variables/(SD/%)	UKB			NHANES			Tianjin		
	No AMD	AMD	*P*	No AMD	AMD	*P*	No AMD	AMD	*P*
*N*	270,297	5,455		3,043	382		564	436	
BMD (BMD_Z)	0.02 (0.98)	−0.19 (0.90)	**<0.001**	1.03 (0.16)	0.94 (0.18)	**<0.001**	1.00 (0.18)	0.86 (0.18)	**<0.001**
Age	56.31 (8.03)	62.72 (5.45)	**<0.001**	56.30 (11.28)	68.70 (11.62)	**<0.001**	58.31 (11.64)	67.36 (11.94)	**<0.001**
Gender			**<0.001**			0.189			**<0.001**
Female	146,600 (54.2)	3,367 (61.7)		1,536 (50.5)	207 (54.2)		222 (39.4)	278 (63.8)	
Male	123,697 (45.8)	2,088 (38.3)		1,507 (49.5)	175 (45.8)		342 (60.6)	158 (36.2)	
Smoke			**<0.001**			**<0.001**			0.239
Never	148,891 (55.1)	2,707 (49.6)		1,462 (48.0)	157 (41.1)		269 (47.7)	201 (46.1)	
Former	93,211 (34.5)	2,255 (41.3)		919 (30.2)	160 (41.9)		177 (31.4)	157 (36.0)	
Current	28,195 (10.4)	493 (9.0)		662 (21.8)	65 (17.0)		118 (20.9)	78 (17.9)	
Alcohol			**<0.001**			**<0.001**			**<0.001**
Never	10,726 (4.0)	298 (5.5)		1,068 (35.1)	165 (43.2)		200 (35.5)	186 (42.7)	
Former	9,240 (3.4)	225 (4.1)		1,084 (35.6)	147 (38.5)		200 (35.5)	170 (39.0)	
Current	250,331 (92.6)	4,932 (90.4)		891 (29.3)	70 (18.3)		164 (29.1)	80 (18.3)	
Education			**<0.001**			**0.049**			
<6th grade	47,081 (17.4)	1,501 (27.5)		354 (11.6)	51 (13.4)				
6th–9th grade	139,606 (51.6)	2,627 (48.2)		447 (14.7)	50 (13.1)				
High school	34,449 (12.7)	578 (10.6)		717 (23.6)	111 (29.1)				
College or above	49,161 (18.2)	749 (13.7)		1,525 (50.1)	170 (44.5)				
BMI	27.41 (4.74)	27.89 (4.85)	**<0.001**	28.24 (5.36)	27.96 (4.71)	0.336	28.03 (5.27)	28.40 (5.29)	0.271
Waist	90.22 (13.39)	91.35 (13.44)	**<0.001**	98.29 (13.61)	100.07 (13.24)	**0.016**			
HBP			**<0.001**			**<0.001**			**<0.001**
No	154,803 (57.3)	2,628 (48.2)		1,577 (51.8)	127 (33.2)		281 (49.8)	167 (38.3)	
Yes	115,494 (42.7)	2,827 (51.8)		1,466 (48.2)	255 (66.8)		283 (50.2)	269 (61.7)	
DM			**<0.001**			0.653			0.843
No	257,302 (95.2)	4,968 (91.1)		2,401 (96.4)	291 (95.7)		547 (97.0)	421 (96.6)	
Yes	12,995 (4.8)	487 (8.9)		89 (3.6)	13 (4.3)		17 (3.0)	15 (3.4)	
HbA1c	35.93 (6.38)	37.64 (8.27)	**<0.001**	5.75 (1.04)	5.76 (0.83)	0.952	5.74 (1.03)	5.75 (0.89)	0.776
RBC	4.50 (0.41)	4.46 (0.40)	**<0.001**	4.70 (0.48)	4.63 (0.52)	**0.011**			
CHOL	5.70 (1.11)	5.69 (1.19)	0.409	5.27 (1.07)	5.19 (1.08)	0.172	5.33 (1.02)	5.24 (1.01)	0.151
ALP	83.32 (25.83)	87.20 (24.10)	**<0.001**	71.43 (23.16)	72.21 (28.17)	0.545	70.45 (19.52)	74.48 (33.04)	**0.016**
ALT	23.53 (13.98)	22.76 (13.43)	**<0.001**	25.98 (21.07)	23.81 (22.77)	0.06	25.08 (14.63)	22.46 (10.47)	**0.002**
AST	26.10 (10.44)	26.29 (9.67)	0.189	26.42 (15.08)	26.52 (17.69)	0.912	26.33 (13.85)	25.33 (7.52)	0.174
GGT	36.93 (41.05)	38.50 (43.45)	**0.005**	33.81 (54.65)	31.07 (49.45)	0.351	33.83 (43.92)	31.54 (46.92)	0.428
CREA	72.13 (17.41)	71.91 (23.71)	0.36	81.89 (40.22)	87.44 (26.13)	**0.009**	84.32 (34.67)	81.54 (26.66)	0.166
URATE	309.52 (78.18)	311.29 (77.16)	0.097	325.85 (82.53)	341.71 (89.94)	**<0.001**	335.48 (81.70)	323.98 (86.74)	**0.032**
Follow_Up	14.13 (0.47)	9.64 (3.35)	**<0.001**						
Glu	5.06 (1.17)	5.26 (1.55)	**<0.001**						
WBC	6.82 (2.10)	6.94 (1.79)	**<0.001**						
PLT	258.15 (59.41)	259.23 (59.71)	0.182						
CRP	2.51 (4.22)	3.13 (5.29)	**<0.001**						
TG	1.75 (1.01)	1.76 (0.95)	0.18						
ALB	45.15 (2.42)	44.66 (2.41)	**<0.001**	42.14 (2.98)	41.75 (3.05)	**0.016**	42.37 (3.08)	41.64 (2.90)	**<0.001**
UREA	5.41 (1.35)	5.66 (1.52)	**<0.001**						
HDL_C				1.39 (0.42)	1.42 (0.44)	0.154	1.37 (0.43)	1.45 (0.43)	**0.003**
PIR				2.92 (1.65)	2.59 (1.41)	**<0.001**			
LYM				2.12 (0.92)	2.03 (0.72)	0.07			
MONO				0.54 (0.18)	0.59 (0.23)	**<0.001**			
NEU				4.17 (1.64)	4.29 (1.62)	0.182			

AMD, age-related macular degeneration; BMD, bone mineral density; BMI, body mass index; HBP, hypertension; DM, diabetes mellitus; HbA1c, glycated hemoglobin; RBC, red blood cell count; CHOL, total cholesterol; ALP, alkaline phosphatase; ALT, alanine aminotransferase; AST, aspartate aminotransferase; GGT, gamma-glutamyl transferase; CREA, creatinine; URATE, uric acid; Glu, fasting glucose; WBC, white blood cell count; PLT, platelet count; CRP, C-reactive protein; TG, triglycerides; ALB, albumin; UREA, blood urea; HDL-C, high-density lipoprotein cholesterol; PIR, poverty income ratio; LYM, lymphocyte count; MONO, monocyte count; NEU, neutrophil count; NHANES, National Health and Nutrition Examination Survey.

Blank cells indicate variables that were not available, not measured, or not harmonized in the corresponding cohort. Because laboratory and socioeconomic variables differed across UK Biobank, NHANES, and the Tianjin cohort, cohort-specific missingness was retained rather than imputed across datasets. Boldface values indicate that the corresponding *P* value is less than 0.05, which is considered statistically significant.

Several demographic and lifestyle factors also differed between cases and controls. Women were more frequently represented among AMD cases in UKB and Tianjin, whereas sex distribution did not differ significantly in NHANES. Former smoking was more common and never smoking less common among cases in UKB and NHANES, while smoking categories were similar in Tianjin. Current drinking was less frequent among cases across all cohorts, and lower education was more common among cases in UKB and NHANES. Hypertension was more prevalent among cases in all 3 cohorts. Diabetes was more frequent among UKB cases but did not differ significantly in NHANES or Tianjin. Anthropometric differences were modest, with higher BMI and waist circumference among UKB cases and higher waist circumference among NHANES cases.

Laboratory measures suggested less favorable metabolic and inflammatory profiles in AMD cases. In UKB, fasting glucose, HbA1c, and C-reactive protein were higher, whereas albumin was lower. Albumin was also lower in AMD cases in NHANES and Tianjin. In UKB, follow-up time was shorter among participants who developed AMD, consistent with event occurrence during follow-up. Overall, AMD cases were characterized by older age, lower BMD, and a greater burden of vascular and metabolic risk factors.

### Observational association analyses across cohorts

**Fig. 2.**
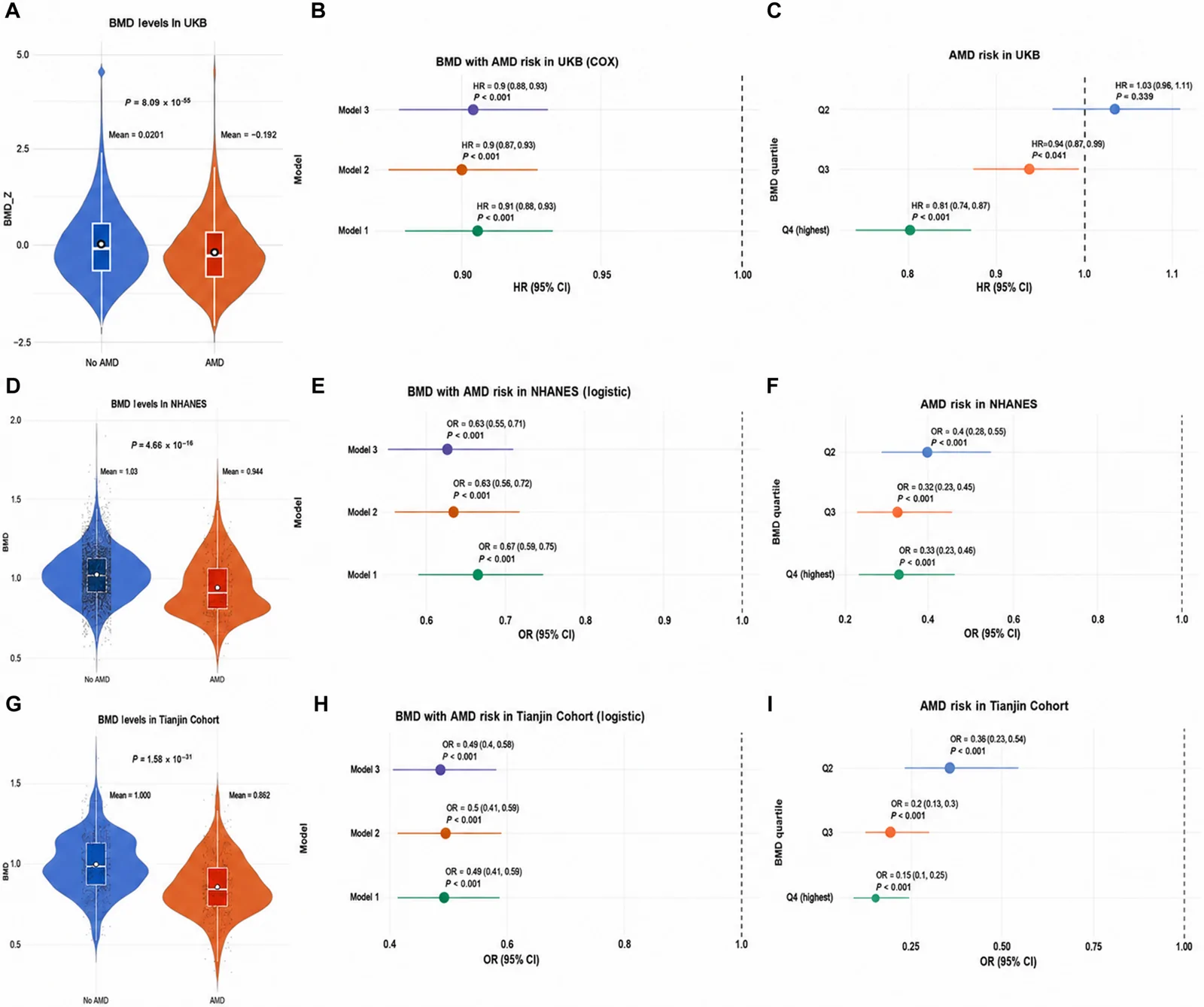
Observational associations between bone mineral density (BMD) and age-related macular degeneration (AMD). (A, D, and G) Violin plots showing lower BMD levels in AMD cases compared with controls in UK Biobank (UKB), National Health and Nutrition Examination Survey (NHANES), and Tianjin cohorts. (B, E, and H) Regression models demonstrating consistent inverse associations between BMD and AMD risk across models with increasing covariate adjustment: hazard ratios (HRs) from Cox models in UKB and odds ratios (ORs) from logistic regression in NHANES and Tianjin. (C, F, and I) Quartile-based analyses showing clear dose-dependent protective gradients of higher BMD against AMD risk in all 3 cohorts. CI, confidence interval.

Across cohorts, BMD distributions were shifted lower in participants with AMD, as shown in the violin plots in Figs. 2A, D, and G. In UKB, Cox models relating baseline BMD to incident AMD yielded hazard ratios of 0.91, 0.89, and 0.88 across models 1 to 3, respectively, all statistically significant (Fig. 2B). In quartile analyses, no difference was observed for Q2, whereas Q3 showed a modest reduction in risk (hazard ratio [HR] ≈ 0.94) and Q4 a larger reduction (HR ≈ 0.81) relative to Q1 (Fig. 2C). In NHANES, logistic regression models yielded odds ratios of 0.69, 0.68, and 0.65 across models 1 to 3 (Fig. 2E), with a graded decrease across quartiles from approximately 0.49 in Q2 to 0.33 in Q4 (Fig. 2F). In Tianjin, the inverse association was stronger, with odds ratios around 0.49 across models and quartile analyses showing a decline from approximately 0.36 in Q2 to 0.20 in Q4 (Figs. 2H, 2I). This stronger effect should be interpreted cautiously because it may reflect clinical diagnosis, DXA-based measurement, hospital-based recruitment, age structure, selection bias, or unmeasured confounding rather than a larger biological effect. Given the substantial differences in cohort design and measurement strategy, formal heterogeneity assessment was performed across cohorts where comparable effect estimates were available, and results were summarized using random-effects models.

Model discrimination increased with additional covariate adjustment. In UKB, AUC rose from 0.742 in model 1 to 0.775 in model 2 and 0.837 in model 3. The corresponding values were 0.783, 0.787, and 0.802 in NHANES and 0.788, 0.792, and 0.806 in Tianjin (Figs. S1A to C). However, these AUC values likely reflect the combined contribution of age, demographic factors, and clinical covariates rather than the independent predictive value of BMD alone. Therefore, AUC results were interpreted as model discrimination for the overall covariate set, while the incremental contribution of BMD was assessed separately using models with and without BMD.

### Nonlinear dose–response and subgroup analyses

**Fig. 3.**
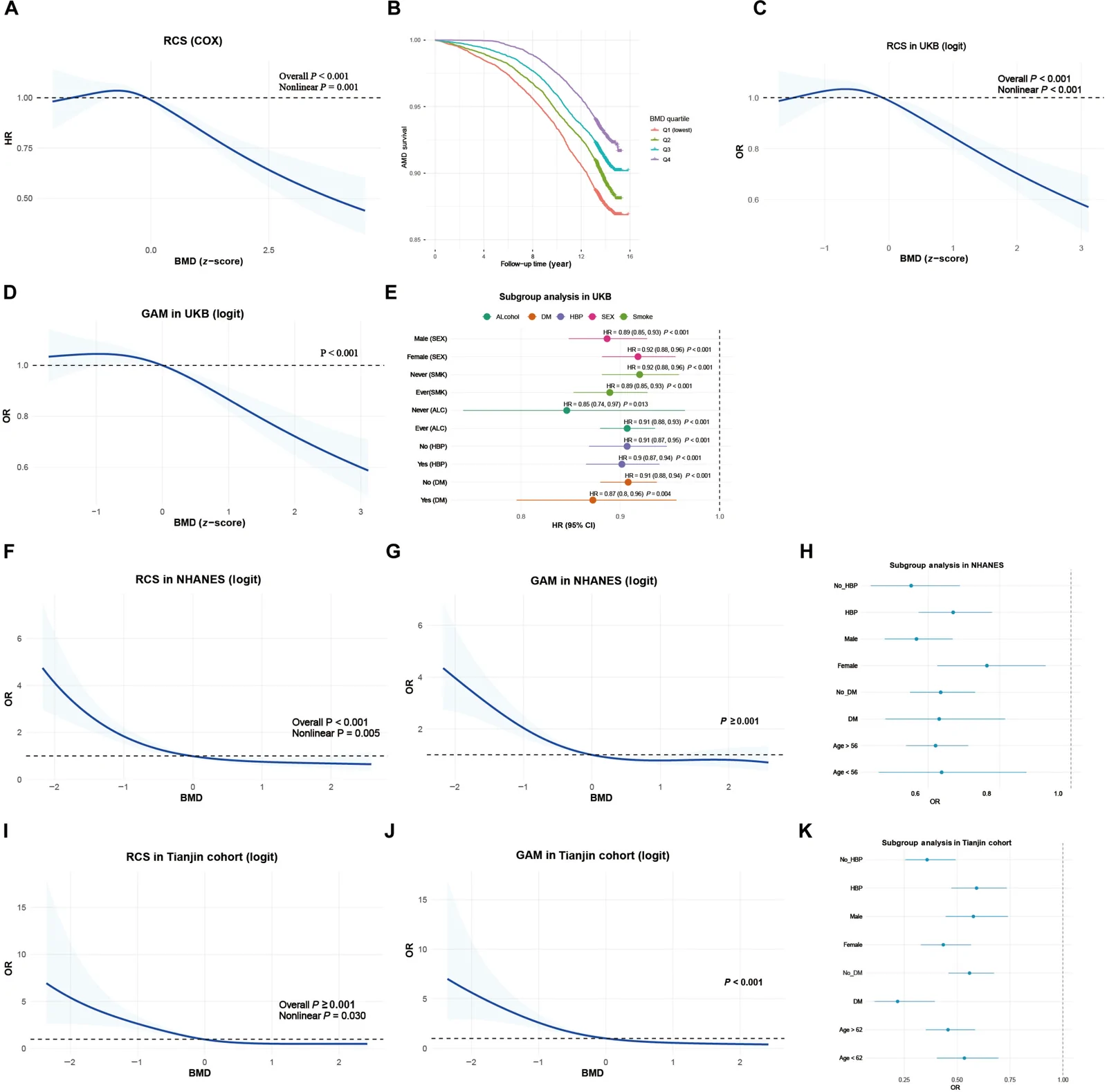
Nonlinear dose–response and subgroup analyses. (A, C, F, and I) Restricted cubic spline (RCS) models showing nonlinear inverse associations between bone mineral density (BMD) and age-related macular degeneration (AMD) risk across UK Biobank (UKB), National Health and Nutrition Examination Survey (NHANES), and Tianjin cohorts. (D, G, and J) Generalized additive models (GAMs) confirming monotonic protective effects of higher bone mineral density (BMD). (B) Kaplan–Meier survival curves in UKB showing separation of age-related macular degeneration (AMD)-free survival by BMD quartiles. (E, H, and K) Forest plots of subgroup analyses indicating consistent associations across sex, age, smoking, alcohol, hypertension, and diabetes.

Higher BMD was associated with lower AMD risk across cohorts, with evidence of nonlinearity. In UKB, RCS analyses within the Cox model showed an inverse relationship, with overall *P* < 0.001 and nonlinearity *P* < 0.001 (Fig. 3A). Logistic spline analyses showed a similar pattern (Fig. 3C), and the GAM indicated a smooth decline in odds with increasing BMD (Fig. 3D). Kaplan–Meier curves stratified by BMD quartiles separated early and remained ordered over time, with the highest AMD-free survival in Q4 (Fig. 3B and Table S2).

In NHANES, spline analyses again supported an inverse relationship, with overall *P* < 0.01 and nonlinearity *P* = 0.005 (Fig. 3F), and the GAM was also significant (*P* < 0.001; Fig. 3G). In Tianjin, spline analyses showed overall *P* < 0.01 and nonlinearity *P* = 0.030 (Fig. 3I), while the GAM yielded *P* < 0.001 (Fig. 3J).

Subgroup forest plots showed generally similar effect estimates across strata defined by sex, age, smoking, alcohol use, hypertension, and diabetes, without strong evidence of effect modification, as shown in Figs. 3E, H, and K (Tables S8 to S10). Correlation chord diagrams summarized the relationships among clinical covariates in UKB, NHANES, and Tianjin, indicating dense interconnections among age, BMD, adiposity, metabolic, hepatic, and renal markers (Tables S1, S3, and S4 and Figs. S2A and B). Mediation analyses suggested that indirect effects from BMD to AMD through individual covariates were generally small, although adiposity- and metabolism-related indicators accounted for the largest proportions in UKB, NHANES, and Tianjin (Figs. S2C to E and Tables S5 to S7).

### Feature selection and risk prediction model

**Fig. 4.**
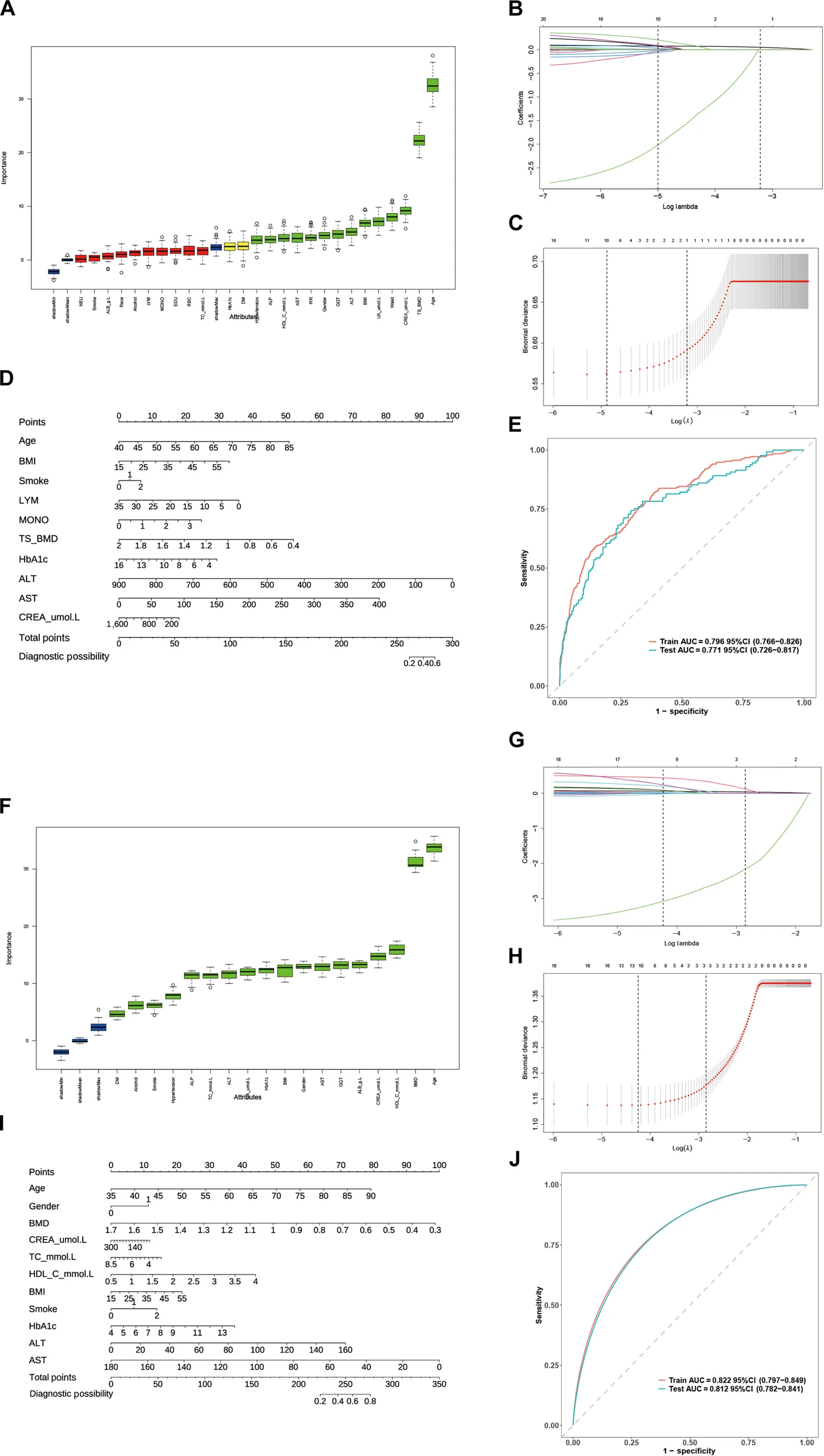
Feature selection and predictive modeling for age-related macular degeneration (AMD). (A and F) Boruta feature selection ranking variables by importance, with age and bone mineral density (BMD) consistently among the top predictors in National Health and Nutrition Examination Survey (NHANES) and Tianjin. (B, C, G, and H) Least absolute shrinkage and selection operator (LASSO) regression paths and deviance curves identifying compact predictive sets. (D and I) Nomograms integrating BMD and other predictors to estimate AMD risk at the individual level. (E and J) Receiver operating characteristic (ROC) curves showing good discrimination in training and test datasets for both NHANES and Tianjin models.

Across datasets, feature selection consistently prioritized age and BMD, with BMI, smoking, and selected hematologic and biochemical variables contributing additional information. In NHANES, Boruta ranked age and BMD among the most important variables (Fig. 4A), and a similar pattern was observed in the Tianjin cohort (Fig. 4F). Cross-validated LASSO identified relatively compact predictor sets, with coefficient paths and deviance profiles shown in Figs. 4B and C for NHANES and Figs. 4G and H for Tianjin.

Using the selected features, we developed nomograms to estimate individual AMD risk, shown for NHANES in Fig. 4D and for Tianjin in Fig. 4I. These nomograms were intended as interpretable summaries of model structure rather than ready-to-use clinical decision tools because they were not externally validated across harmonized populations and did not define clinical screening thresholds or downstream referral strategies. Model discrimination was acceptable in both cohorts. In NHANES, the ROC analysis showed an AUC of 0.796 in the training set and 0.771 in the test set (Fig. 4E). In Tianjin, the corresponding AUCs were 0.892 and 0.812 (Fig. 4J). These results indicate that cohort-specific models incorporating age, BMD, and a limited set of additional variables achieved acceptable discrimination within their respective datasets. Because AMD ascertainment, BMD modality, covariate availability, and population structure differed across cohorts, these analyses should be interpreted as cohort-specific modeling with recurrent feature patterns rather than as external validation of a single unified prediction model.

### Machine learning classification and interpretability

**Fig. 5.**
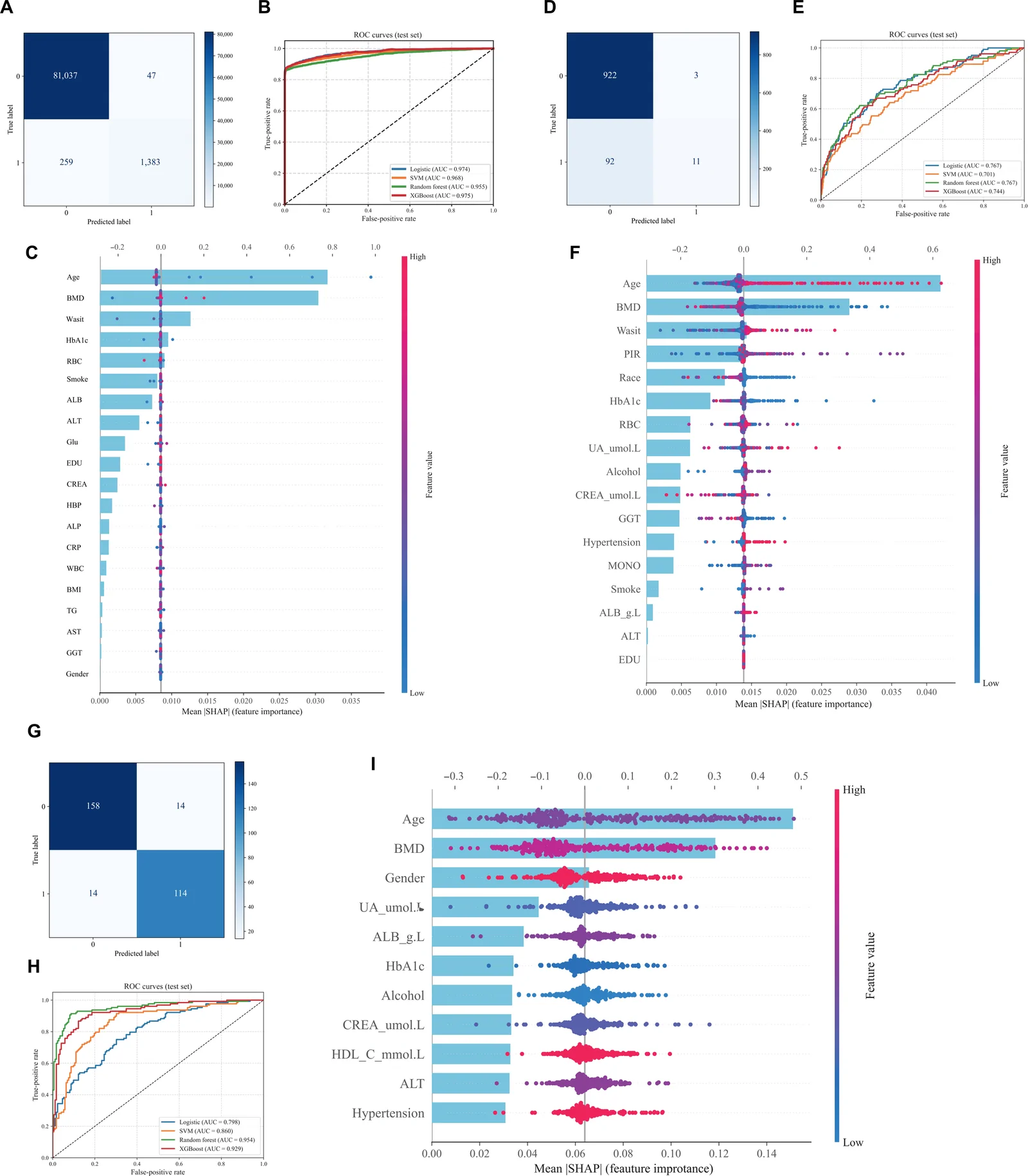
Machine learning classification and interpretability. (A, D, and G) Confusion matrices of the best-performing classifiers in UK Biobank (UKB), National Health and Nutrition Examination Survey (NHANES), and Tianjin. (B, E, and H) receiver operating characteristic (ROC) curves comparing performance of multiple machine learning algorithms, highlighting extreme gradient boosting (XGBoost) as best in UKB, logistic regression in NHANES, and random forest in Tianjin. (C, F, and I) SHapley Additive exPlanation (SHAP) summary plots showing feature importance across models, consistently placing age and bone mineral density (BMD) as the top contributors, with additional influences from metabolic and biochemical traits.

In UKB, XGBoost achieved the highest discrimination among the evaluated algorithms in this cohort-specific analysis. Its confusion matrix showed high specificity with balanced sensitivity (Fig. 5A), and SHAP-based global importance ranked age and BMD highest, followed by waist circumference, HbA1c, red blood cell count, urate, and albumin (Figs. 5B and C). In NHANES, logistic regression yielded the highest ROC AUC; its confusion matrix showed balanced classification performance (Fig. 5D), the ROC comparison confirmed its relative advantage over alternative models (Fig. 5E), and SHAP analyses again highlighted age and BMD, together with waist, poverty income ratio, race, HbA1c, red blood cells, uric acid, alcohol use, creatinine, and gamma-glutamyl transferase (Fig. 5F). In the Tianjin cohort, random forest achieved the highest ROC AUC among the evaluated algorithms, although this should be interpreted as cohort-specific performance rather than evidence that random forest is generally superior. SHAP analyses identified age and BMD as major predictive contributors, along with sex, uric acid, albumin, HbA1c, alcohol use, creatinine, HDL cholesterol, alanine aminotransferase, and hypertension.

Additional results are shown in the supplementary figures. In UKB, confusion matrices for the other algorithms and a radar chart summarizing accuracy, precision, recall, AUC, specificity, and predictive values are presented in Figs. S3A to D. In NHANES, alternative models are summarized in Figs. S6A to D, while SHAP dependence plots in Fig. S4 indicate increasing risk with age and decreasing risk with higher BMD; other metabolic and hematologic variables showed smaller and mostly monotonic effects. SHAP interaction plots in Fig. S5 suggested generally weak pairwise interactions without a dominant cross-term. In Tianjin, alternative classifiers are summarized in Figs. S9A to D. SHAP dependence plots in Fig. S7 again showed a negative contribution from BMD and a positive contribution from age, while Fig. S8 suggested only modest pairwise interactions among metabolic variables.

### MR analysis

**Fig. 6.**
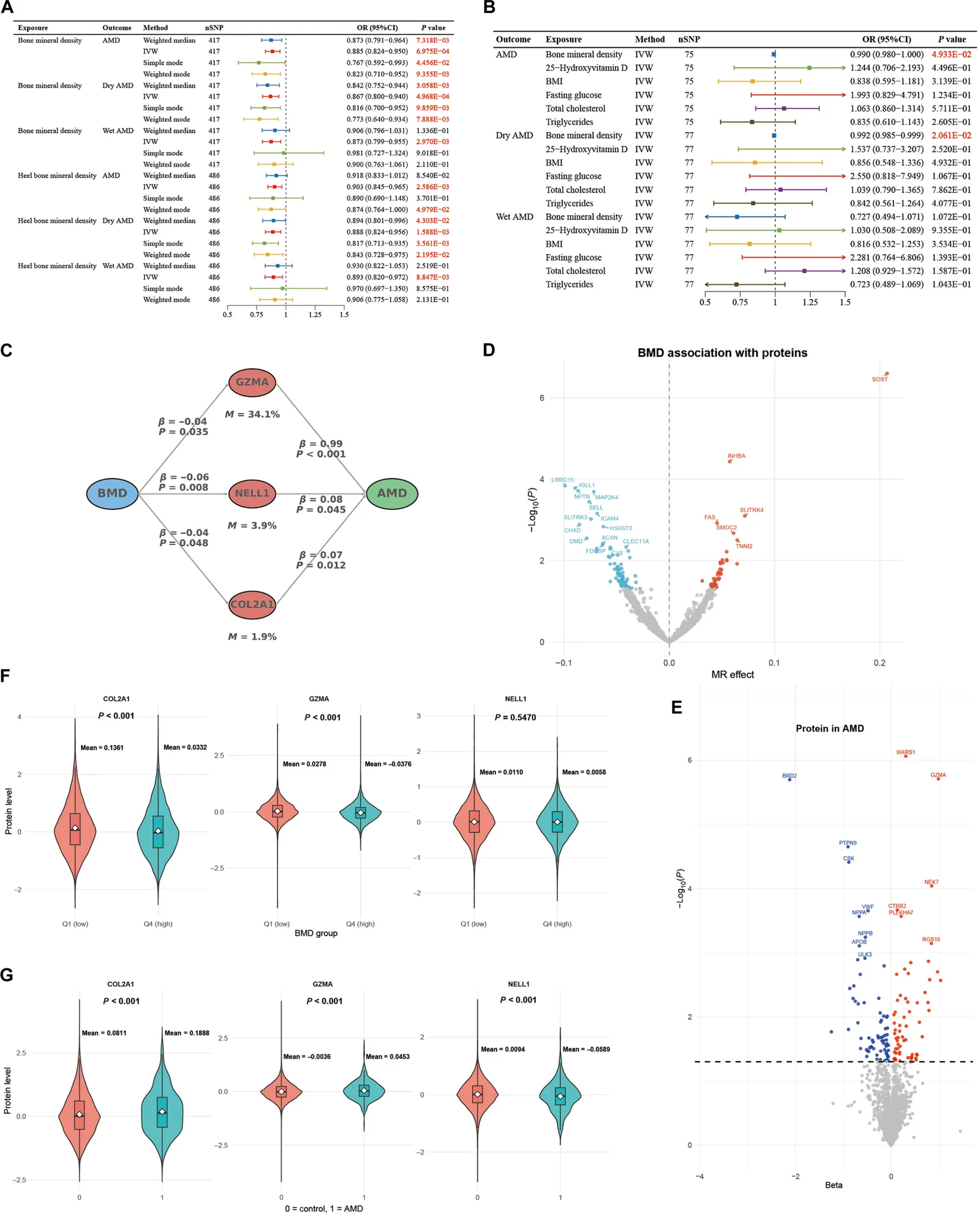
Mendelian randomization analyses linking bone mineral density (BMD) to age-related macular degeneration (AMD) and candidate mediating proteins. (A) Two-sample Mendelian randomization (MR) showing protective causal effects of higher BMD on overall, dry, and wet AMD across multiple estimators. (B) Multivariable MR confirming independent effects of BMD after adjustment for vitamin D, body mass index (BMI), glucose, and lipid traits. (C) Mediation MR schematic identifying granzyme A (GZMA), NEL-like protein 1 (NELL1), and collagen type II alpha 1 chain (COL2A1) as potential intermediates between BMD and AMD. (D and E) Volcano plots of MR effects of BMD on proteins and proteins on AMD, highlighting overlapping candidates. (F) Violin plots showing lower GZMA and COL2A1 in high-BMD individuals. (G) Violin plots showing higher levels of GZMA, NELL1, and COL2A1 in AMD cases compared with controls.

Two-sample MR analyses provided supportive evidence consistent with a potential inverse contribution of genetically predicted BMD to AMD risk across multiple instruments and estimators. Using body BMD and heel BMD as exposures, IVW estimates for overall AMD were below unity, and the direction of effect was generally consistent across weighted median, weighted mode, and simple mode analyses. Similar trends were observed for both dry and wet AMD, as summarized in Fig. 6A. In multivariable MR, jointly modeling BMD with vitamin D, BMI, fasting glucose, total cholesterol, and triglycerides, BMD remained independently associated with lower risk, particularly for overall AMD and the dry subtype (Fig. 6B). Scatter plots further illustrated the negative slopes for body BMD to overall AMD, heel BMD to overall AMD, and each BMD measure to dry and wet AMD (Figs. S10A to F). Reverse-direction analyses did not support an effect of genetically predicted AMD on BMD, and tests for horizontal pleiotropy and heterogeneity were largely unremarkable across overall, dry, and wet AMD analyses (Tables S11 to S16).

To explore molecular pathways compatible with mediation, 2-step MR prioritized 3 circulating proteins—granzyme A (GZMA), NEL-like protein 1 (NELL1), and collagen type II alpha 1 chain (COL2A1)—as candidate molecular intermediates linking BMD-related genetic signals and AMD risk. These proteins should be interpreted as prioritized circulating markers with mediation-compatible evidence rather than as established mechanistic mediators. The schematic in Fig. 6C shows negative genetic effects from BMD to these proteins and positive effects from these proteins to AMD, with approximate mediation proportions of one-third for GZMA and smaller proportions for NELL1 and COL2A1. The volcano plot of MR effects from BMD to proteins is shown in Fig. 6D, and the corresponding protein-to-AMD associations are shown in Fig. 6E. Orthogonal validation in UKB proteomics was directionally supportive. Individuals in the highest BMD quartile had lower plasma GZMA and COL2A1 than those in the lowest quartile, whereas NELL1 did not show an evident BMD gradient (Fig. 6F). In case-control analyses, AMD cases had higher levels of GZMA, NELL1, and COL2A1 than controls (Fig. 6G).

Fig. S13 summarizes metabolite pathway enrichment from MR analyses across 2 links: BMD to metabolites and metabolites to AMD. Panels A and B show pathways enriched among metabolites decreasing or increasing with higher BMD, including aromatic amino acid metabolism, sulfur handling, nucleotide transport and salvage, mitochondrial energy–nitrogen pathways, and small-molecule transport. Panels C and D show pathways enriched among metabolites associated with lower or higher AMD risk. Protective signals clustered around short-chain fatty acid and amino acid biosynthesis/conjugation, whereas risk-associated signals were enriched in lysine and aromatic amino acid turnover, redox/cofactor metabolism, and ubiquinone-related pathways. Dot size represents enrichment ratio and color indicates significance. Overall, these analyses suggest that amino acid, nucleotide, and energy metabolism may contribute to the bone–eye axis.

### Proteomic and metabolomic association analyses

**Fig. 7.**
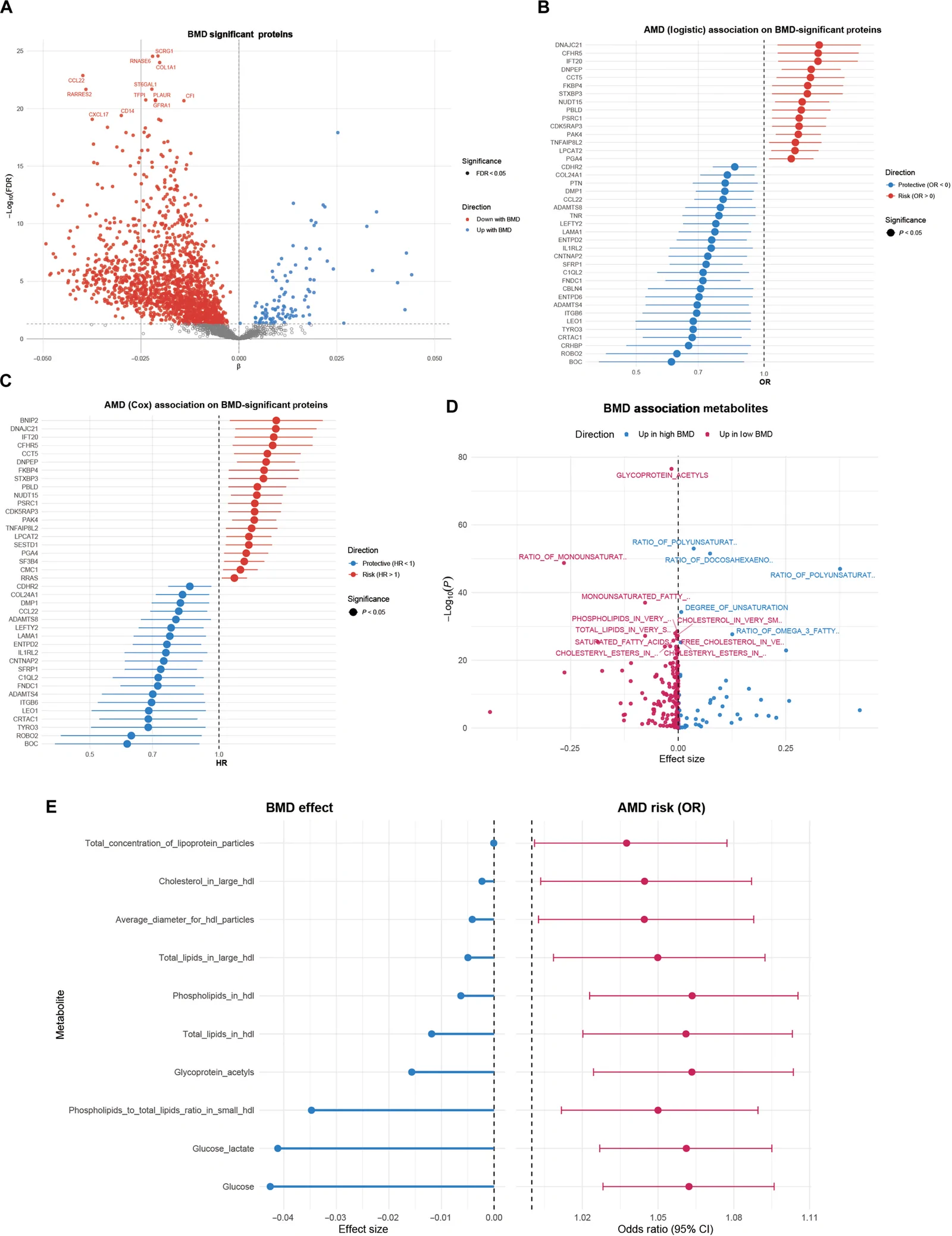
Proteomic and metabolomic association analyses. (A) Volcano plot of bone mineral density (BMD)-associated proteins, enriched for extracellular matrix components. (B and C) Logistic and Cox regression analyses showing significant associations of BMD-linked proteins with age-related macular degeneration (AMD) status and incidence. (D) Volcano plot of BMD-associated metabolites highlighting lipid and inflammatory features, including high-density lipoprotein (HDL) particle traits and glycoprotein acetyls. (E) Integrated comparison showing overlapping metabolites that decrease with higher BMD but increase with higher AMD risk.

Within the UKB omics subset, multiple circulating proteins varied across BMD levels, and a subset was also associated with AMD. The volcano plot of BMD-associated proteins showed a broad distribution, with many proteins lower at higher BMD; highlighted proteins included extracellular-matrix-related molecules such as COL2A1 and regulators including NELL1 and lumican (Fig. 7A and Tables S17 and S19). When these BMD-significant proteins were tested against AMD, several showed positive associations in cross-sectional logistic regression models, whereas others showed inverse associations (Fig. 7B). Similar patterns were observed in prospective Cox analyses for incident AMD (Fig. 7C and Tables S18 and S20). Heatmaps stratified by BMD quartiles and AMD status further visualized these gradients and suggested coherent expression blocks across both logistic-selected and Cox-selected protein panels (Figs. S11 and S12).

Metabolomic analyses showed a complementary pattern. The volcano plot of BMD-associated metabolites highlighted lipid- and inflammation-related signals, including HDL particle traits and glycoprotein acetyls, which tended to be lower in individuals with higher BMD (Fig. 7D). Integrated analyses indicated that several metabolites reduced at higher BMD were also associated with higher AMD odds, including HDL-related measures and glycoprotein acetyls (Fig. 7E). Together, these data suggest that extracellular matrix remodeling, lipid transport, and systemic inflammatory pathways may represent shared molecular features associated with lower BMD and higher AMD risk. However, HDL-related measures and glycoprotein acetyls have complex and context-dependent roles in cardiovascular, inflammatory, and retinal disease. Therefore, these metabolomic findings should be interpreted as pathway-level associations rather than as direct evidence of a unified lipid-inflammatory mechanism.

### Retinal and functional alterations in low-BMD rat model

**Fig. 8.**
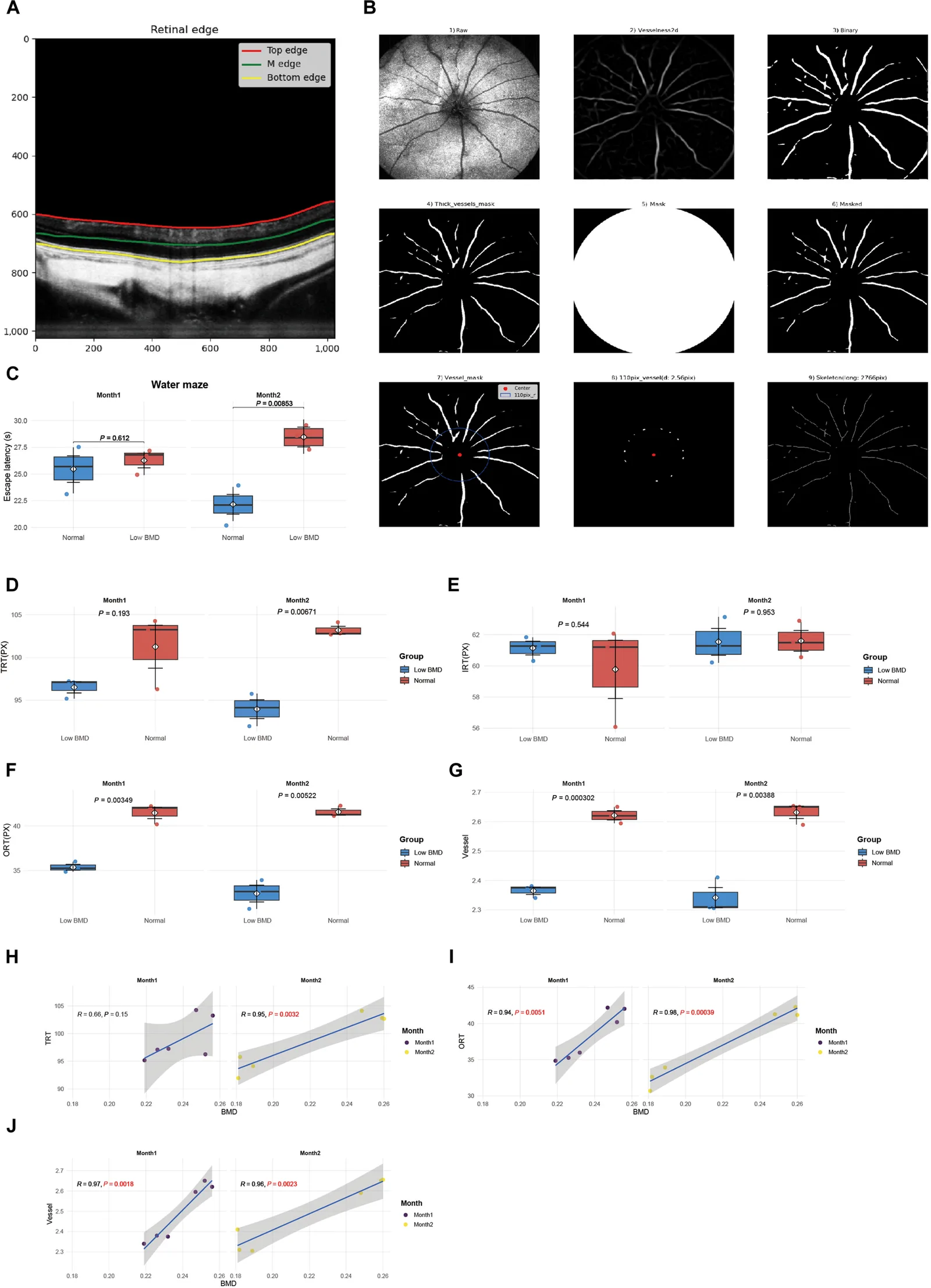
Retinal and functional alterations in the low-bone mineral density (BMD) rat model. (A) Optical coherence tomography (OCT) cross-sections of the rat retina with clear delineation of inner and outer retinal layers. (B) Image processing pipeline for retinal vasculature quantification. (C) Water maze escape latency showing significant visual–spatial impairment in low-BMD rats at month 2. (D to F) Retinal thickness analyses showing progressive reduction in total and outer retinal thickness in low-BMD rats. (G) Retinal vessel caliber significantly narrowed in low-BMD rats at both month 1 and month 2. (H to J) Correlation analyses demonstrating positive associations between BMD and total retinal thickness, outer retinal thickness, and vessel caliber, especially at month 2.

Quantitative retinal imaging and vessel extraction were established using a standardized pipeline, as shown in Figs. 8A and B. Water maze performance was similar between groups at month 1 but differed at month 2, when escape latency was longer in the low-BMD group (*P* < 0.01; Fig. 8C), suggesting delayed visual–spatial performance.

Retinal structural analyses showed selective thinning. Total retinal thickness did not differ significantly at month 1 but was lower in the low-BMD group at month 2 (*P* < 0.01; Fig. 8D). Inner retinal thickness did not differ at month 1 and showed a borderline reduction at month 2 (*P* near 0.05; Fig. 8E). Outer retinal thickness was reduced at month 1 (*P* < 0.05) and remained lower at month 2 (*P* < 0.01; Fig. 8F). Retinal vessel caliber was smaller in low-BMD animals at both time points (*P* < 0.001 at month 1 and *P* < 0.01 at month 2; Fig. 8G).

Correlation analyses showed that higher BMD was associated with greater total retinal thickness, greater outer retinal thickness, and wider vessel caliber, with stronger relationships at month 2; at that time point, coefficients of determination were high and all *P* values were < 0.01 (Fig. 8H to J). Overall, the glucocorticoid-induced low-BMD model was associated with delayed visual–spatial performance, outer retinal thinning, and narrower retinal vessels. However, the model did not reproduce hallmark features of human AMD, such as drusen, retinal pigment epithelium dysfunction, complement deposition, Bruch membrane alteration, geographic atrophy, or choroidal neovascularization. These findings therefore support nonspecific retinal alterations under low-bone-mass conditions rather than validation of AMD-like pathology.

## Discussion

This study provides multilayered evidence that lower BMD is associated with higher AMD risk across datasets with different designs, phenotype definitions, and measurement strategies. UKB supports a prospective association with incident AMD, NHANES supports an imaging-based cross-sectional association, and the Tianjin cohort provides hospital-based clinical replication. These evidence sources are complementary but not directly equivalent. The observed association demonstrated nonlinear dose–response patterns and persisted after multivariable adjustment, although residual confounding, age-related biological processes, and measurement heterogeneity remain important considerations. MR analyses provided supportive genetic evidence directionally consistent with a potential inverse contribution of higher genetically predicted BMD to AMD risk, but these findings should not be interpreted as definitive causal proof. Proteomic and metabolomic analyses identified overlapping molecular signatures involving extracellular matrix remodeling, lipid transport, amino acid metabolism, and inflammation. Together, these findings support a systemic-aging-related framework linking skeletal health and retinal vulnerability, while stopping short of establishing a direct one-to-one causal bone–eye pathway.

AMD has well-established genetic and ocular mechanisms involving complement factor H, age-related maculopathy susceptibility 2, HtrA serine peptidase 1, complement C3, complement C2, complement factor B, cholesteryl ester transfer protein, TIMP metallopeptidase inhibitor 3, COL8A1, apolipoprotein E, complement activation, Bruch membrane biology, retinal pigment epithelium lipid handling, and choroidal vascular regulation. Our study does not replace these canonical AMD pathways. Instead, the BMD-associated signals identified here should be viewed as systemic-aging-related correlates that may interact with, modify, or reflect susceptibility to these eye-specific mechanisms. Future studies integrating ocular imaging phenotypes, retinal or retinal pigment epithelium (RPE)-specific molecular data, and genetic risk scores will be needed to clarify how systemic skeletal aging relates to canonical AMD biology.

A central contribution of this study lies in its integration of observational, predictive, and causal inference frameworks within a unified analytical structure. These approaches address fundamentally different questions and should be interpreted accordingly. Observational analyses quantify associations at the population level, machine learning models evaluate predictive performance, and MR analyses provide evidence toward causality under specific assumptions. Importantly, predictive importance does not imply causal relevance. The consistent ranking of BMD alongside age in machine learning models indicates its contribution to classification within the dataset, whereas MR findings suggest that genetically proxied BMD may have a modest causal relationship with AMD. Taken together, these results position BMD not as a dominant driver but as a systemic marker embedded within interconnected aging-related biological networks.

The nonlinear relationship observed between BMD and AMD risk provides additional biological insight. Across cohorts, the protective effect of higher BMD plateaued beyond a certain range, suggesting threshold or saturation dynamics. This pattern may be interpreted within the hypothesis of physiological reserve, in which structural integrity and functional resilience operate within constrained biological limits. Beyond these limits, further increases in BMD may confer diminishing apparent benefit. This interpretation remains hypothesis generating, and the present data do not directly prove that BMD itself determines systemic resilience or retinal pathology.

Multiomics integration further strengthens this system-level interpretation. Proteomic analyses highlighted extracellular matrix components and regulatory proteins jointly associated with BMD and AMD, while metabolomic profiling implicated lipid transport, amino acid metabolism, and inflammatory pathways [34]. These findings converge on shared biological axes—including chronic low-grade inflammation, oxidative stress, and extracellular matrix remodeling—that are not organ specific but operate across multiple tissues during aging [35,36]. Within this framework, BMD may be conceptualized as an integrative phenotype capturing cumulative endocrine, metabolic, and immune dysregulation, which, in turn, influences retinal vulnerability [37].

The experimental findings provide supportive, although not definitive, biological context. In the glucocorticoid-induced low-BMD rat model, we observed early retinal alterations, including outer retinal thinning, vascular narrowing, and impaired visual behavior. These features are consistent with early or nonspecific degenerative processes affecting retinal structure and microvasculature [38]. However, the model does not reproduce hallmark pathological features of human AMD, such as drusen formation or advanced atrophy [39]. Therefore, these results should be interpreted as demonstrating AMD-relevant retinal alterations rather than direct evidence of AMD pathogenesis [40]. The experimental data support the plausibility of a systemic link but do not establish a direct mechanistic pathway.

From a translational perspective, the distinction between statistical association and clinical utility is critical. Although BMD was consistently associated with AMD risk and contributed to model performance, its incremental predictive value beyond age and other established risk factors appears modest. This reflects a broader principle in predictive modeling: Variables that are statistically robust in large datasets may contribute limited additional discrimination when dominant predictors are already included. Nevertheless, BMD remains a clinically accessible and objective measure that may capture aspects of biological aging not fully represented by traditional risk factors. Its potential value may therefore lie in integration within multiparameter risk stratification frameworks rather than as a standalone predictor.

The consistency of findings across heterogeneous cohorts strengthens the robustness of the conclusions while also highlighting important methodological considerations. Differences in AMD ascertainment and BMD measurement modalities across datasets may introduce heterogeneity and potential misclassification. However, the convergence of results across independent populations, analytical approaches, and biological layers suggests that the observed association reflects an underlying biological signal rather than cohort-specific artifacts.

Despite these strengths, several limitations should be acknowledged. First, AMD ascertainment differed across cohorts, including medical records and self-report in UKB, retinal imaging in NHANES, and clinical diagnosis in the Tianjin cohort; therefore, these datasets should be interpreted as complementary rather than fully harmonized AMD phenotypes. Second, BMD measurement was not identical across datasets, with heel-ultrasound-derived estimated BMD in UKB and DXA-based measurements in NHANES and Tianjin. Although BMD was standardized within each cohort, differences in skeletal site, measurement principle, and error structure may affect cross-cohort comparability. Third, residual confounding cannot be excluded. Age, frailty, menopause-related factors, vascular aging, healthcare contact, medication use, supplementation, ophthalmic examination frequency, and genetic susceptibility may influence both BMD and AMD but were not fully captured across all cohorts. Fourth, model 3 should be interpreted cautiously because some biochemical variables may represent downstream systemic markers rather than pure confounders. Thus, the fully adjusted model was used as a broad sensitivity analysis rather than a definitive causal estimate. Fifth, the predictive modeling analyses were exploratory. Although BMD contributed to model-based risk classification, its incremental clinical utility beyond age and established risk factors requires further validation using models with and without BMD, calibration assessment, decision curve analysis, and external validation. Sixth, MR provides supportive genetic evidence but cannot definitively prove causality. Horizontal pleiotropy, sample overlap, ancestry-specific effects, weak instrument bias, and complex genetic architecture may still influence the estimates. Seventh, the proteomic and metabolomic analyses were mainly observational and largely based on UKB subsets. Therefore, these findings should be interpreted as shared molecular signatures and pathway-level hypotheses rather than direct evidence of mechanistic mediation. Eighth, the animal experiment provides supportive biological context but does not validate AMD pathology. The glucocorticoid-induced low-BMD rat model had a small sample size, short follow-up, and did not reproduce hallmark AMD features such as drusen, RPE dysfunction, Bruch membrane alteration, geographic atrophy, or choroidal neovascularization. Finally, this study does not replace canonical AMD mechanisms involving complement activation, ARMS2/HTRA1, CFH, RPE lipid handling, Bruch membrane biology, and choroidal vascular regulation. Future studies should integrate harmonized longitudinal AMD phenotyping, standardized BMD measurement, ocular imaging biomarkers, eye-specific molecular profiling, and larger experimental models to clarify the temporal and biological relationship between skeletal aging and retinal degeneration.

## Conclusion

In conclusion, this study shows that lower BMD is associated with higher AMD risk across 3 complementary datasets and provides supportive genetic and molecular evidence consistent with shared systemic-aging-related pathways. Because AMD ascertainment, BMD measurement, cohort structure, and covariate availability differed across datasets, these findings should be interpreted as convergent but heterogeneous evidence rather than definitive proof of a direct causal bone–eye pathway. BMD may serve as an accessible associated marker of biological aging and retinal vulnerability, but its incremental clinical utility for AMD risk prediction requires further validation using harmonized phenotypes, formal incremental prediction analyses, calibration assessment, and external validation. Future studies should integrate longitudinal imaging, multiomics profiling, eye-specific molecular data, and mechanistic experiments to clarify the temporal and biological relationship between skeletal aging and AMD.

## Ethical Approval

All human analyses were conducted in accordance with the Declaration of Helsinki and the governance frameworks of each contributing cohort. The hospital-based Tianjin cohort and related measurements were reviewed and approved by the Medical Ethics Committee of Tianjin Eye Hospital, approval number KY-2023WJW003, and all participants provided written informed consent. The UKB obtained ethics approval and written informed consent from all participants, and data use in this study adhered to the resource terms of access. NHANES 2005 to 2008 datasets are deidentified and publicly available; original protocols received national ethics oversight with written informed consent, and the secondary use of deidentified data in this study complied with institutional requirements. All animal procedures were approved by the Institutional Animal Care and Use Committee of Tianjin Eye Hospital and adhered to established welfare guidelines.

## Data Availability

UK Biobank data are available upon approved application to the UK Biobank. The NHANES 2005–2008 datasets are publicly accessible from the US Centers for Disease Control and Prevention. Summary statistics for genome-wide association analyses were derived from large-scale resources, including body bone mineral density and heel bone mineral density, age-related macular degeneration, proteome-wide protein quantitative trait loci from deCODE, and metabolome-wide genome-wide association studies from the Canadian Longitudinal Study on Aging. The Tianjin cohort data and all animal experiment measurements are available from the corresponding author upon reasonable request, provided that institutional approvals for data use have been obtained. The analysis code and key scripts used to generate figures and tables will be made available by the corresponding author upon request. The data and materials used to support the findings of this study are available from the corresponding author upon reasonable request.
