# Supplementary material for: Decoding the Bone–Eye Axis: Machine Learning for Age-Related Macular Degeneration Risk Prediction
Source: Cyborg Bionic Syst. 2026 Sep 14;7:0676. doi: 10.34133/cbsystems.0676 (PMC13572803; doi:10.34133/cbsystems.0676)
Supplement: Supplementary file 1 [file cbsystems.0676.f1.zip › Supplementary Materials.docx]

**Figure S1. ROC curve comparisons across models.**

(A) UK Biobank models with different covariate adjustments, showing improved discrimination from Model 1 (AUC=0.742) to Model 3 (AUC=0.837).

(B) ROC curves in NHANES models, with incremental improvements up to Model 3 (AUC=0.802).

(C) ROC curves in the Tianjin cohort, showing consistent increases up to Model 3 (AUC=0.806).

**Figure S2. Correlation and mediation analyses of clinical variables.**

(A, B) Chord diagrams displaying correlations among clinical and biochemical variables in NHANES and Tianjin cohorts.

(C–E) Mediation analyses estimating indirect effects of selected variables in UKB, NHANES, and Tianjin cohorts, suggesting partial contributions of adiposity, glycemic and metabolic indicators to the BMD–AMD relationship.

**Figure S3. Confusion matrices and performance radar plot for UKB models.**

(A–C) Confusion matrices for logistic regression, random forest, and XGBoost classifiers in UKB, showing varying sensitivity and specificity.

(D) Radar plot comparing accuracy, precision, recall, specificity, AUC, F1 score, and predictive values across four machine learning methods, with XGBoost showing the best balanced performance.

**Figure S4. SHAP dependence plots for NHANES model.**

Dependence plots illustrating marginal effects of top predictors including age, BMD, waist circumference, HbA1c, RBC count, albumin, GGT, creatinine, uric acid, HDL-C, alcohol intake, hypertension, and smoking on AMD risk, with nonlinear trends for BMD and age most prominent.

**Figure S5. SHAP interaction plots for NHANES model.**

Pairwise interaction plots showing combined effects of age, BMD, waist, PIR, race, HbA1c, RBC, uric acid, alcohol, creatinine, GGT, hypertension, albumin, ALT, and EDU. Interactions highlight additive and modest synergistic effects, particularly for age–BMD and BMD–waist pairs.

**Figure S6. Confusion matrices and radar plot for NHANES models.**

(A–C) Confusion matrices for logistic regression, random forest, and XGBoost classifiers in NHANES, reflecting different balances of sensitivity and specificity.

(D) Radar chart comparing overall accuracy, precision, recall, specificity, AUC, and predictive values, showing logistic regression as top performer in this dataset.

**Figure S7. SHAP dependence plots for Tianjin cohort model.**

Dependence plots for top predictors, showing negative contributions of higher BMD and positive contributions of higher age and HbA1c to AMD risk, with additional roles of ALT, albumin, creatinine, uric acid, HDL-C, alcohol use, hypertension, and gender.

**Figure S8. SHAP interaction plots for Tianjin cohort model.**

Pairwise SHAP interaction plots indicating relationships between age, BMD, gender, albumin, HbA1c, alcohol, creatinine, uric acid, HDL-C, ALT, and hypertension, highlighting combined nonlinear effects, particularly between BMD and age or gender.

**Figure S9. Confusion matrices and radar plot for Tianjin models.**

(A–C) Confusion matrices for different classifiers showing varying classification accuracy.

(D) Radar chart comparing accuracy, recall, precision, specificity, AUC, F1 score, and predictive values, with random forest performing best in this cohort.

**Figure S10. Mendelian randomization scatter plots of BMD and AMD.**

(A, C, E) Scatter plots for body BMD with overall, dry, and wet AMD as outcomes.

(B, D, F) Scatter plots for heel BMD with overall, dry, and wet AMD as outcomes.

Lines represent causal estimates from inverse variance weighted, weighted median, weighted mode, simple mode, and MR-Egger methods, consistently supporting protective effects of higher BMD on AMD risk.

**Figure S11. Heatmap of logistic-significant proteins.**

Heatmap showing expression patterns of proteins significantly associated with AMD in logistic regression models, clustered by sex, BMD quartiles, and AMD status. Distinct protein modules were identified, with higher or lower expression patterns in low-BMD individuals and AMD cases, supporting shared proteomic pathways.

**Figure S12. Heatmap of Cox-significant proteins.**

Heatmap displaying proteins significantly associated with incident AMD in Cox regression models, annotated by sex, BMD quartiles, and AMD status. The clustering highlights proteins enriched in individuals with low BMD and higher AMD risk, reflecting proteomic links between skeletal and retinal degeneration.

**Figure S13. Metabolite set enrichment analyses.**

(A, B) Enrichment analysis of metabolites negatively or positively associated with BMD in MR analyses, highlighting amino acid, nucleotide, and energy metabolism pathways.

(C, D) Enrichment analysis of metabolites negatively or positively associated with AMD, showing clustering in amino acid turnover, oxidative stress, and lipid-related pathways. Collectively, these results reveal metabolic signatures connecting bone mineral density with AMD risk.
